# “I agree with LGBT rights, but…”: Authoritarianism and social dominance orientation underlying hypocritical attitudes of Taiwan society

**DOI:** 10.3389/fpsyg.2022.1062748

**Published:** 2022-12-22

**Authors:** Han-Yu Hsu

**Affiliations:** School of Social Development, East China University of Political Science and Law, Shanghai, China

**Keywords:** hypocrisy, right wing authoritarianism, same sex marriage, social dominance orientation, prejudice

## Abstract

In the modern public sphere, ordinary people may display hypocrisy in political participation, showing contradictory attitudes across different social issues. But there still exists another type of hypocritical attitude within one single issue, such as agreeing with LGBT rights but refusing to amend the current Civil Code simultaneously in the case of Taiwan. In the same-sex marriage legalizing process, the hypocritical attitude could be observed in Taiwan’s conservative campus, together with the explicitly prejudiced attitude. In this article, we explored the existence of the hypocritical attitude on this issue and discovered its psychological foundations. We conducted an online questionnaire survey in 2018 (*N* = 544) to measure Taiwanese participants’ attitudes toward same-sex marriage and their psychological dispositions of Right-Wing Authoritarianism (RWA) and Social Dominance Orientation (SDO). Our results showed that while attitudes toward LGBT rights and special-law were negatively correlated, several participants showed the hypocrisy of positive attitudes toward the two sets of questions simultaneously. The hypocritical people shared similar psychological dispositions with the explicitly prejudiced people as high in RWA and SDO while differentiated from the LGBT-friendly people. Attitudinal hypocrisy and explicit prejudice constitute two sides of the conservative camp in Taiwan, which is based on the Confucianism cultural value of interpersonal harmony. The cultural and societal implications were discussed.

## 1. Introduction

On May 24, 2019, same-sex marriage finally became legal in Taiwan by the *Act for Implementation of Judicial Yuan Interpretation No. 748* (“Same-Sex Marriage in Taiwan,” 2020). Taiwan’s legalization process of same-sex marriage experienced nearly 30 years of social movement, and the social controversy became highly intense since the passing of *Judicial Yuan Interpretation No.748* on May 24, 2017 (Judicial Yuan, 2017; Liu, 2017). The intensity of the controversy was reflected in the referendum in November 2018. Three conservative proposals about same-sex marriage were adopted, one limiting marriage of Civil Code between man and woman (No.10), one banning gender equality education in elementary and middle school (No. 11), and one asking the *Interpretation No.748* should not be implemented by amending the Civil Code (No. 12). Two progressive ones were vetoed, one demanding to amend the Civil Code to ensure same-sex marital rights (No. 14), and one demanding to do gender equality education in elementary and middle school (No. 15, Central Election Commission, 2021).

Although several Christian groups in the conservative camp proposed the anti-same-sex marriage questions, Christianity is not the predominant religion in Taiwan (Liu, 2010), resulting in a different cultural context of same-sex marriage compared to the west. Meanwhile, the traditional Chinese Confucianism culture is influential in Taiwan, especially in the conservative camp, which highly values interpersonal harmony (Hwang, 2012; Huang, 2016). Thus, some conservatives in Taiwan are authentically homophobic, while others, driven by the motivation of interpersonal harmony of traditional values, are just neglecting the controversy and chaos from the amendment of existing law. The latter’s attitudes towards LGBT people could be expressed as positive about maintaining interpersonal harmony and avoiding interpersonal conflict with the pro-LGBT people, while their defending attitude towards the existing law system lets them be the *de facto* alliance with the LGBT-prejudiced group, which is defined as hypocrisy (Collins, 2018).

Past research has demonstrated that there are certain psychological dispositions underlying individual’s politically conservative attitudes (e.g., Duckitt, 2001; Jost et al., 2003), including Right-Wing Authoritarian (RWA, Altemeyer, 1996) and Social Dominance Orientation (SDO, Pratto et al., 1994). Meanwhile, little research differentiated explicit prejudice and hypocritical attitudes and explored their psychological foundations. In the present article, we will employ the questionnaire method to explore the existence of attitudinal hypocrisy on the issue of same-sex marriage in Taiwan. Also, we will use RWA and SDO as psychological indexes of conservatism and test their functional similarities and differences in predicting prejudiced and hypocritical attitudes in the current issue.

### 1.1. Explicit prejudice and hypocrisy: Two sides of conservative attitudes towards same-sex marriage

In political psychology, RWA and SDO are regarded as significant psychological predictors of conservative attitudes (Wilson and Sibley, 2013), including explicit prejudice. RWA refers to individuals’ authoritarianism (Altemeyer, 1996), and high-RWA people prefer social conformity and are generally more religious, which would be more sex-prejudiced (Herek and McLemore, 2013). Meanwhile, SDO refers to individuals’ desire for their in-group as the superior, dominating group (Pratto et al., 1994), and high-SDO people would favor hierarchy-enhancing ideologies and policies more, increasing their prejudice toward sex minority people (Pratto et al., 1994, 1997). In empirical studies, RWA and SDO could predict an individual’s conservative stands on explicit sex prejudice and anti-same-sex marriage attitudes (Koleva et al., 2012; Poteat and Mereish, 2012; Hsu et al., 2019).

In the case of Taiwan, the society is highly influenced by Confucianism culture (Hwang, 2012), and there may exist another type of conservative attitude along with explicit prejudice, which we call hypocrisy. Compared to fundamentalist Judaism, Christianity, and Islam which condemn homosexual behaviors as sinful (Herek and McLemore, 2013), Confucianism does not regard homosexuality as a sin, nor do Taoism or Buddhism. Meanwhile, it highly values interpersonal harmony and regards interpersonal conflicts as negative events (Huang, 2016), encouraging individuals’ conflict-avoidant behaviors in public opinion expression. In the current case, mainstream public opinion’s acceptance of same-sex relations increases yearly along with the democratization process and LGBT rights movements (Lee, 2006). Driven by the cultural motivation of interpersonal harmony and conflict avoidance, some conservatives in Taiwan may express positive attitudes toward LGBT rights to avoid potential conflicts with other people. Since political conservatism is characterized by maintaining the status quo and conserving the existing social institutions (Jost et al., 2003), these conservatives would disagree with changing the existing legal system while avoiding expressing explicit prejudice towards LGBT rights.

Such an attitudinal and behavioral pattern is in accordance with hypocrisy, defined as an individual’s attitude-behavior or attitude-attitude inconsistency (Collins, 2018). In empirical studies, attitudinal hypocrisy is operationalized as the inconsistency across attitudes along one particular ideology (Abrams et al., 2015; Collins, 2018). Suppose we regard the attitudinal spectrum from pro-LGBT to anti-LGBT as an ideology. In that case, the prementioned conservative attitude is a kind of hypocrisy for its inconsistency along this axis.

The actual political behaviors before and during the 2018 referendum revealed the existence of hypocrisy. The anti-LGBT camp proposed the No. 12 question, “do you agree to implement the protection of same-sex couples’ rights of permanent cohabitation by ways other than amending the marriage definition in the Civil Code” (Central Election Commission, 2021). This question seemed to agree with protecting LGBT rights at first glance. Still, it also made the same-sex union differentiated from the Civil Code marriage, increased the risk of virtual discrimination, and was criticized by the pro-same-sex marriage camp. Hypocrisy in public opinion functions as a *de facto* obstruction to legalizing same-sex marriage.

So, are the conservatives more hypocritical, or do liberals also have their hypocrisy in different types? Although the word hypocrisy is often used to attack political opponents by both conservatives and liberals (Collins, 2018), whether hypocrisy correlates with a particular ideology is still in dispute in social science. For example, Collins (2018) argued that both liberals and conservatives have their hypocrisy in different types. On the contrary, some researchers argued that high-RWA people are more self-contradictory thinking (Altemeyer, 2007), and high-SDO people tend to be more cynical and double-minded (Radkiewicz and Skarżyńska, 2021), both of which would increase hypocrisy. In an integrated model of conservatism, Jost et al. (2003) argued that psychological conservatives need more for social order and tolerate less ambiguity, which may influence hypocrisy in two different paths. When someone searches for stable social order, they may tend to avoid interpersonal conflicts, which may undermine orderliness, resulting in more hypocrisy. On the other path, when someone searches for cognition closure to avoid ambiguity, they may behave less hypocritically. These two paths could reduce conflicts and uncertainty; the former is interpersonal, while the latter is intrapersonal.

In the case of Taiwan society, the traditional Confucian culture highly values interpersonal harmony and regards interpersonal conflicts as negative events (Huang, 2016). In empirical studies on authenticity (defined as inconsistency of behavior, as opposed to hypocrisy), it has been found that East Asians show less consistency across contexts (English and Chen, 2007; Slabu et al., 2014). Considering the close relationship between traditionalism and conservatism (Jost et al., 2009; Duckitt and Bizumic, 2013), it would be possible that conservatives in Taiwan, who value tradition more than liberals, would be more hypocritical because of the greater need for interpersonal harmony and avoidance of interpersonal conflict, which would be examined in the current research.

### 1.2. Current research

In this research, we focused on ordinary people’s attitudes toward the legalization of same-sex marriage and examined the effects of psychological traits on it. To measure the attitudes, we designed two sets of questionnaires. The first three items were the general supportiveness for legalizing same-sex marriage. The second three items, derived from the conservative camp’s proposal No.12 in the 2018 referendum and related narrative, were the pro-special-law attitude that prefers to legist a special law for the demand of Interpretation No. 748 while maintaining the status quo of the Civil Code. The wording of items was shown in the next Method part. Based on the definition of hypocrisy and the theoretical background, we proposed three hypotheses:

*H1*: There exists a part of participants with a hypocritical attitude that supports LGBT rights while opposing changing the current law.

*H2*: The people with hypocritical and prejudiced attitudes would be more psychologically conservative (higher RWA and SDO) compared to the pro-LGBT side.

## 2. Materials and methods

### 2.1. Participants

A total of 544 Taiwanese participated in our research, comprising 270 women (49.6%), 268 men (49.3%), and 6 others (1.1%). Their mean age was 30.71 years (standard deviation [*SD*] = 9.29 years). Education levels (including ongoing ones) were 3 with less than high school (0.6%), 51 with high school (0.1%), 377 with college and undergraduate (69.3%), and 113 with postgraduate or above (20.8%).

### 2.2. Materials and procedure

We recruited participants online through Facebook and other forums from January to April 2018. Participants were invited to complete an anonymous online questionnaire that lasted 10–15 min. The informed consent was provided on the first page of the online questionnaire, and each participant was free to quit at any stage. No rewards were provided. All questions were presented in Chinese. This study was approved by the Research Ethics Committee of National Taiwan University. Data were analyzed using the SPSS version 25 package.

### 2.3. Attitudes towards same-sex marriage

According to the social situation at the time of data collection, we designed two sets of questionnaires: the first three items for the supportiveness of same-sex marriage and the last three items for the pro-Special-Law attitude on this issue. Since the Chinese participants are more likely to choose the middle point as a special kind of social desirability (Yang, 2001), the items were measured by a 6-point scale ranging from −3 to 3 without a 0. The English translation of these items is presented in Table 1. The scores of the two sets had high internal consistency.

**Table 1:** Items of attitudes to same-sex marriage.

Items	*M*(*SD*)
Supportiveness of same-sex marriage: (*α* = 0.93)	
1. Homosexual people should have the same rights to pursue their own happiness as heterosexuals.	2.29(1.22)
2. Homosexual people should have the right to marry.	2.10(1.49)
3. I agree with legalizing same-sex marriage by the amendment of the Civil Code.	1.89(1.77)
*Pro-Special-Law* attitude: (*α* = 0.78)	
4. I prefer legalizing same-sex marriage by creating a new special law rather than amending the Civil Code.	−1.01(2.12)
5. The homosexual people’s right to happiness could be pursued without the necessity of marriage institution in law.	−1.38(1.92)
6. Homosexual people could have their own love affair, but it’s unnecessary to amend everyone’s law system.	−1.69(1.87)

### 2.4. Right-wing authoritarianism

We adopted Huang’s (2007) Chinese translation of RWA. In Huang’s research, the RWA questionnaire showed a three-factor structure in the Taiwanese context: one positive factor considering tradition/conservation, and two negative factors of openness and autonomy. In consideration of the length of the questionnaire, we selected three items with the largest factor loadings from the positive factor tradition/conservation to represent the construct of RWA in the Taiwanese context. The items were measured by a 6-point scale ranging from −3 to 3 without a 0 (*α* = 0.87).

### 2.5. Social dominance orientation

In consideration of the length of the questionnaire, we selected the five items of the Chinese version of the SDO with the largest loadings in Huang’s (2007) study and measured them on a 6-point scale ranging from −3 to 3 without a 0 (*α* = 0.82).

## 3. Results

### 3.1. Descriptive statistics

The means, *SD*s, and correlations of RWA, SDO, and attitudes are shown in Table 2. The supportiveness of same-sex marriage and the maintaining-status-quo attitude were negatively correlated, indicating that most people have a consistent attitude on the same-sex issue. RWA and SDO were negatively correlated with the supportiveness of same-sex marriage and positively with the maintaining-status-quo attitude, which was consistent with our previous research (Hsu et al., 2019).

**Table 2:** Descriptive statistics of right-wing authoritarianism (RWA), social dominance orientation (SDO), and attitudes to same-sex marriage.

	*M*(*SD*)	1	2	3
1 RWA	−0.78(1.71)			
2 SDO	−1.13(1.23)	0.64^**^		
3 Supportiveness of same-sex marriage	2.09(1.42)	−0.62^**^	−0.42^**^	
4 Pro-Special-Law attitude	−1.36(1.64)	0.72^**^	0.54^**^	−0.58^**^

** *p* < 0.01 (two-tailed).

To discover the distribution of attitudes toward same-sex marriage and to check if there were people with hypocritical attitudes, we conducted a cluster analysis of the two attitudinal scores on same-sex marriage (3 and 4 in Table 2). A two-step strategy in SPSS 25.0 was employed. The method with Bayesian Information Criterion and Akaike’s Information Criterion produced an identical three-cluster solution with a.70 silhouette measure of cohesion and separation, indicating a stable and fair result (Sarstedt and Mooi, 2014).

To describe the properties of the three clusters, we tested their difference on the four main measurements in our study. As in Table 3, Cluster 1 (*N* = 69) did not support same-sex marriage (*M* = −1.00, *t*_*M*–0_ = −7.94, *p* < 0.01), while they showed a neutral stand on special law (*M* = 0.15, *t*_*M*–0_ = 1.08, *p* = 0.28). Cluster 2 (*N* = 150) supported the same-sex marriage in general (*M* = 1.68, *t*_*M*–0_ = 27.28, *p* < 0.01), but they also preferred the special law with no risk for amending the existing Civil Code (*M* = 0.43, *t*_*M*–0_ = 5.07, *p* < 0.01). Cluster 3 explicitly supported the same-sex marriage (*M* = 2.94, *t*_*M*–0_ = 247.14, *p* < 0.01) while opposing the special law (*M* = −2.50, *t*_*M*–0_ = −66.28, *p* < 0.01), indicating their support to amend the Civil Code. Thus, we named Cluster 1 as Prejudice, Cluster 2 as Hypocrisy, and Cluster 3 as LGBT-friendly, whose attitudes on same-sex marriage were differentiated from each other in *F*-test. Our hypothesis 1 was supported.

**Table 3:** Right-wing authoritarianism (RWA), social dominance orientation (SDO), and attitudinal differences among clusters.

Measurements	*M* (*SD*)			*F-*test
	Cluster 1 Prejudice *N* = 69	Cluster 2 Hypocrisy *N* = 150	Cluster 3 LGBT-friendly *N* = 325	
Supportiveness of same-sex marriage	−1.00 (1.04)	1.68 (0.75)	2.94 (0.21)	1431.80^**^ 1 < 2 < 3
Pro-special-law attitude	0.15 (1.15)	0.43 (1.03)	−2.50 (0.68)	720.17^**^ 3 < 1 < 2
RWA	1.05 (1.10)	0.64 (1.30)	−1.83 (1.06)	342.80^**^ 3 < 2 < 1
SDO	−0.34 (1.04)	−0.27 (1.13)	−1.69 (0.96)	124.29^**^ 3 < 1 = 2

The post-hoc tests were conducted by the LSD method at.05 level.

** *p* < 0.01 (two-tailed).

In addition, the political-psychological traits of the three clusters were also significantly different. The LGBT-friendly people were the most liberal cluster with the lowest RWA and SDO (LSD: *p*s < 0.01). The Prejudice people’s RWA was higher than Hypocrisy people (LSD: *p* < 0.01). At the same time, their SDOs were not significantly different (LSD: *p* = 0.61). Our hypothesis 2 was supported.

### 3.2. Predicting hypocrisy by logistic regression

We conducted multi-nominal logistic regression on the three cluster types to test whether RWA, SDO, and demographic variables could explain the individual difference in hypocrisy tendency. Including the two dummy variables of gender (gender_men and gender_other, with women as referencing group) caused the singularity warning in logistic regression in SPSS for only 6 participants who selected “other” in the gender question. Since the women and other gender showed no differences in the two sets of same-sex attitudes (*t*_1_ = −0.75, *p*_1_ = 0.46; *t*_2_ = 0.84, *p*_2_ = 0.40), we combined these two groups and compared them with men in logistic regression. The results are shown in Table 4.

**Table 4:** Multi-nominal logistic regression on three clusters.

IV\DV	Cluster 1: Prejudice reff: LGBT-friendly			Cluster 2: Hypocrisy reff: LGBT-friendly			Cluster 2: Hypocrisy reff: Prejudice		
	*B*	*p*	OR	*B*	*p*	OR	*B*	*p*	OR
Intercept	**−6.05**	**<0.01**		**−2.04**	**0.02**		**4.01**	**<0.01**	
Gender_men reff: women and other	**2.69**	**<0.01**	**14.78**	**1.40**	**<0.01**	**4.07**	−1.29	0.10	0.28
Age	**0.12**	**<0.01**	**1.13**	**0.07**	**<0.01**	**1.07**	**−0.06**	**<0.01**	**1.15**
Education	−0.30	0.39	0.74	−0.16	0.60	0.86	0.14	0.55	0.93
RWA	**0.90**	**<0.01**	**2.47**	**0.83**	**<0.01**	**2.30**	−0.07	0.63	1.13
SDO	0.38	0.06	1.47	**0.51**	**<0.01**	**1.67**	0.13	0.42	0.28
−2 Log likelihood	538.54								
*χ* ^2^	*χ*^2^_(10)_ = 462.76, *p* < 0.01								
Pseudo *R*^2^	Cox and Snell: 0.57; Nagelkerke: 0.68; McFadden: 0.46								

The first two equations’ reference category was LGBT-friendly (Cluster 3), while the third was Prejudice (Cluster 1). Significant coefficients at.05 level were in bold characters.

In the first and second equations, men, elders, and high-RWA participants were more likely to be Prejudiced and Hypocrisy. High-SDO participants were more likely to be hypocritical compared to the LGBT-friendly cluster. In the third equation comparing Prejudice with Hypocrisy, only age could slightly decrease the possibility of being hypocritical, while their RWA and SDO did not differentiate. In sum, psychologically conservative people (high-RWA and high-SDO) were more likely to be explicitly prejudiced or hypocritical. Our hypothesis 2 was supported.

## 4. Discussion

Previous researchers have pointed out that hypocrisy is both a kind of discourse to accuse opponents and an individual’s behavior pattern driven by specific psychological mechanisms (Collins, 2018). In this research, we found the existence of a hypocritical attitude in the current issue of same-sex marriage legalization in Taiwan, along with explicit prejudiced and LGBT-friendly attitudes. We also found that the male, elder, high-RWA, and high-SDO people are more likely to hold a hypocritical attitude towards the same-sex marriage issue than to be LGBT-friendly. In contrast, the differentiation between hypocritical and explicit prejudiced attitudes could only be predicted by age with a relatively small effect size.

According to these results, attitudinal hypocrisy could be considered conservative at individual and societal levels. In view of individual differences, the hypocritical people shared similar psychological dispositions (high RWA and SDO) but differentiated from LGBT-friendly people. At the societal level, although hypocritical people may express their support for LGBT rights in some situations, their superficial support would not be transferred to the social pressure for institutional progress, such as the amendments of laws, being a *de facto* alliance with the conservatives with explicit prejudice.

The current research focused on the same-sex marriage issue in the social context of Taiwan. At the same time, we anticipate similar attitudinal hypocrisy might also exist on other issues and social contexts. Besides, two theoretical questions could be derived from the current study, which are discussed below.

### 4.1. Why is the “un-integrated” hypocritical cognition possible?

Hypocrisy is defined as inconsistency between attitude/behavior or attitude/attitude. Its existence implies that individuals can hold logically inconsistent cognition schema, which contradicts classical psychology theories about cognition integration. In cognition dissonance theory (Festinger, 1957) and other mainstream psychology theories, the integration of cognition is a basic psychological need, triggering individuals’ psychological stress when facing contradiction. It is also the mechanism that raising awareness of hypocrisy could reduce behavior inconsistency in moral hypocrisy studies (e.g., Stone et al., 1997). But according to our result, many participants hold inconsistent hypocritical attitudes.

So, why is the “un-integrated” cognition of hypocrisy possible? We agree that each person needs a certain level of cognition integration, while we argue that hypocrisy in social attitudes does not necessarily mean unintegrated cognition. In a constructivist view, different people organize their cognition by different standards. The hypocritical individuals just integrate their cognition and behaviors according to other standards instead of the logical relationship between/within social issue narratives. In the current case of Taiwan, a solid cultural standard of interpersonal harmony exists, which conservatives advocate as a traditional value. Under certain conditions, the principle to ensure interpersonal harmony would overcome the need for logical consistency of attitude expression. Thus, the hypocritical individuals’ cognition is not un-integrated in actuality; it is integrated by other principles instead of the issue’s content *per se*. Theoretically, the inconsistency between attitude/behavior or attitude/attitude can be differentiated with cognition integration; the former is about interpersonal inconsistency, and the latter is intrapersonal.

The current research takes Confucianism as a cultural context to analyze the conservatives’ hypocritical tendency, driven by the cultural motivation of interpersonal harmony and conflict avoidance (Huang, 2016). It does not mean that this kind of attitudinal hypocrisy only exists in Confucianism cultural context. Instead, we anticipate that there would be other cultural standards in non-Confucianism societies, which have a similar function as interpersonal harmony.

Nevertheless, in the current empirical study, we did not directly measure the need for interpersonal harmony or conflict avoidance by cultural psychological scales. In future research, the measurements of cultural values could be adapted to explore the mechanism of hypocrisy from the view of individual differences. From the perspective of social cognition, the schema of interpersonal harmony and conflict could also be explored by designing priming methods, which may also contribute to changing the hypocritical tendency in certain situations. Other cultural and social factors in non-Confucian contexts could also be explored.

Along this vein, there is another question that could not be solved in the current article. Do these hypocritical people agree with LGBT rights authentically, or are they pretending to be LGBT-friendly according to the “political correctness” of modern egalitarian values, but indeed homophobic? Since we only used explicit measurements by questionnaire method, our result could not differentiate these two kinds of individuals. Further research could employ implicit measurement tools to identify these two kinds and discover their psychological and ideological dispositions.

### 4.2. The relationship between conservatism and hypocrisy

Previous research disputed whether conservatives are more hypocritical. For example, Nam et al. (2013) proposed that conservatives avoided dissonance-arousing situations more to ensure their cognition consistency, while Collins (2018) found that both conservatives and liberals showed hypocrisy in America. In this vein, our study provided evidence in Taiwan that the psychological conservatives (indexed by high RWA and SDO) were more likely to be hypocritical.

Back to the basic psychological mechanism, Jost et al. (2003) proposed an integrated motivation model of conservatism. In this model, conservatives are more sensitive to uncertainty and threat, resulting in more need for cognition closure and social order. The need for cognition closure is about intrapersonal consistency and conflict, which may inhibit conservatives’ hypocrisy, while the need for social order is about interpersonal harmony and conflict, which may facilitate conservatives’ hypocrisy. We infer that these two components of conservatism are the reason for previous inconsistent research results.

In our research, as indexes of conservatism, both RWA and SDO could increase the likelihood of being hypocritical or prejudiced, compared to being LGBT-friendly. In the case of Taiwan, the traditional Confucianism value, including interpersonal harmony, plays as a cultural system that would interact with the social system, including conservatism. Just as the social-cultural interaction covered in analytical dualism theory (Archer, 1995), this interaction may result in the conservatives’ hypocritical tendency in our research. Future research should explore the contradiction between keeping internal cognition consistent and assuring interpersonal order systematically by cross-culture and cross-societies samples. Cultural variables should be included to quantitatively study how the culture and social systems interacted at the individual level.

### 4.3. Limitations

The current article also has some limitations. When designing the questionnaire, we did not measure participants’ sexual orientation, which may relate to their attitude to same-sex marriage. We asked participants’ self-identified gender, and 6 participants (1.1%) chose other gender instead of man or woman. But the frequency was too small, and including the variable of gender_other into the logistic regressions would result in errors in statistics software. Future research should use a larger sample to test the gender and sexual orientation’s effects on hypocritical attitudes.

For the psychological measurements, we used RWA and SDO as indexes of conservatism. Still, we did not directly measure the cultural-psychological variables to confirm the relationship between conservatism and the value of interpersonal harmony. Besides, non-Confucianism cultures may have their factors to promote or restrain hypocritical behaviors. Further research could take a cross-cultural perspective to systematically discover the underlying cultural and social mechanism of hypocrisy.

When operationalizing hypocrisy, we employed cluster analysis to check whether a certain number of participants simultaneously had a positive attitude toward LGBT rights and a positive attitude toward special law. The cluster analysis to group the participants will definitely lose information about individual differences within each group. Rigorous speaking, it could only give a qualitative operationalization of hypocrisy, but not a quantitative operationalization of an individual’s hypocrisy tendency with a range. Previous research provided other methods to define hypocrisy quantitatively, such as the within-individual standard deviance of a set of attitudes along an ideology axis (see Collins, 2018, p. 39–44). But in the current research, the two sets of questions of pro-LGBT rights and pro-special-law were not entirely logically reversed, and even the pro-LGBT people could agree with the special law to a certain extent as a tactic of compromise with the conservative public. Thus, we did not use Collins’ approach to calculate hypocrisy. Further research should design questions along one axis with logical consistency and investigate the extent of hypocrisy by quantitative operationalization.

## Data availability statement

The dataset of this article is available from the corresponding author, xuhanyu@ecupl.edu.cn, upon reasonable request.

## Ethics statement

The studies involving human participants were reviewed and approved by Research Ethics Committee of National Taiwan University. Written informed consent for participation was not required for this study in accordance with the national legislation and the institutional requirements.

## Author contributions

The author confirms being the sole contributor of this work and has approved it for publication.

## Conflict of interest

The author declares that the research was conducted in the absence of any commercial or financial relationships that could be construed as a potential conflict of interest.

## Publisher’s note

All claims expressed in this article are solely those of the authors and do not necessarily represent those of their affiliated organizations, or those of the publisher, the editors and the reviewers. Any product that may be evaluated in this article, or claim that may be made by its manufacturer, is not guaranteed or endorsed by the publisher.
